# Real-world treatment outcomes and adverse events during BPaLM therapy for rifampicin-resistant TB

**DOI:** 10.5588/ijtldopen.26.0269

**Published:** 2026-09-15

**Authors:** C.V. Choong, E.P. Soh, Y.Y. Ong, J.X. Tay, C.H.L. Khoong, A.E.L. Lau, C.B.E. Chee

**Affiliations:** 1 National Tuberculosis Care Centre, Tan Tock Seng Hospital, Singapore;; 2 Department of Respiratory and Critical Care Medicine, Tan Tock Seng Hospital, Singapore;; 3 Pharmacy Division, National Centre of Infectious Diseases, Tan Tock Seng Hospital.

**Keywords:** tuberculosis, Singapore, drug toxicity, MDR-TB, linezolid

## Abstract

**BACKGROUND:**

Clinical trials have demonstrated high efficacy of bedaquiline, pretomanid, and linezolid with or without moxifloxacin (BPaLM/BPaL) for rifampicin-resistant and multidrug-resistant TB (RR/MDR-TB), but real-world implementation data remain limited, particularly from South-East Asian programmatic settings. We report Singapore’s national experience with BPaLM/BPaL, focusing on treatment outcomes and adverse drug events.

**METHODS:**

We conducted a retrospective analysis of all RR/MDR-TB patients treated with BPaLM/BPaL at the National Tuberculosis Care Centre (NTBCC), Singapore, between July 2022 and September 2025. Data on demographics, microbiology, treatment characteristics, adverse drug reactions, and outcomes were extracted from a standing database.

**RESULTS:**

Thirty-three patients were included (median age 39 years, 45.4% men). Over half were asymptomatic at diagnosis, and 90.9% had culture-confirmed disease. All patients who completed treatment locally achieved treatment success without any relapses observed during available follow-up. Adverse drug reactions requiring regimen modification occurred in 21 patients (63.6%). Moxifloxacin was discontinued for 14 patients (42.4%), predominantly due to QTc prolongation and gastrointestinal intolerance while 11 patients (33.3%) required linezolid dose reduction due to haematological toxicity and peripheral neuropathy.

**CONCLUSIONS:**

BPaLM/BPaL achieved excellent early effectiveness but required frequent regimen modifications. Successful implementation of short-course RR/MDR-TB regimens requires anticipatory toxicity monitoring, flexible regimen management, and robust treatment support systems.

TB remains a global public health emergency, with an estimated global total of 10.7 million people who fell ill with TB in 2024.^1^ Before 2022, drug-resistant TB, particularly rifampicin-resistant and multidrug-resistant (RR/MDR) TB, posed a disproportionate threat to TB control due to its prolonged treatment duration, higher costs, increased drug toxicity, and poorer treatment outcomes compared with drug-susceptible TB.^2^ In recent years, the management of RR/MDR-TB has undergone a paradigm shift. Since 2018, the WHO has recommended 9-month all-oral regimens for the treatment of MDR/RR-TB, replacing injectable-containing regimens associated with significant toxicity.^3^ Subsequent updates to WHO guidelines in 2022 and 2024 incorporated evidence from pivotal clinical trials, including NIX-TB,^4^ ZeNIX^5^ and TB-PRACTECAL,^6^ which demonstrated that a 6-month treatment of bedaquiline, pretomanid, and linezolid with or without moxifloxacin (BPaLM/BPaL) achieved high treatment success rates ranging from 85% to 93%, with fewer adverse events than conventional long or 9-month regimens.^7^

Although these trials established the efficacy of BPaLM/BPaL regimens under trial conditions, real-world implementation data remain limited and is mostly derived from heterogeneous observational cohorts and early implementation cohorts.^8–12^ There remains limited programmatic experience describing adverse event patterns, regimen modifications, and operational challenges in routine clinical care, especially within the South-East Asian population, where patient comorbidity and operational constraints may differ from trial and European and African programme settings.

Following the 2022 WHO recommendations,^13^ the National Tuberculosis Care Centre (NTBCC) in Singapore adopted BPaLM/BPaL for the treatment of patients with RR/MDR-TB. As the national referral centre for TB, NTBCC manages all cases of RR/MDR-TB in Singapore, providing a unique opportunity to examine real-world implementation of these regimens within a national TB programme. We report our programmatic experience with BPaLM/BPaL, focusing on treatment outcomes, adverse events patterns, drug-specific regimen adaptations, and operational challenges encountered during routine clinical implementation.

## METHODS

We conducted a retrospective cohort analysis of patients treated with BPaLM/BPaL at NTBCC, Singapore, between 1 July 2022 and 21 September 2025. The eligibility criteria for BPaLM/BPaL regimen analysis included patients with confirmed RR either through genotypic testing or phenotypic testing. Data were extracted from a standing database maintained at the NTBCC which retrospectively captures patients’ demographics, clinical characteristics, drug-susceptibility testing results, regimen details, adverse events, culture conversion status, and final treatment outcomes. Culture conversion was defined as time from treatment started to second consecutive negative cultures taken at least 30 days apart. Treatment success was defined as successfully completed treatment without bacteriological evidence of failure, as per the WHO.^14^

### Statistical analysis

Categorical variables were compared using Fisher’s exact test due to small sample size. Continuous variables were expressed as median (interquartile range [IQR]) and compared using the Mann–Whitney *U* test. A two-sided *P* value of <0.05 was considered statistically significant. Analyses were performed using Jamovi (version 2.3).

### Ethical statement

This study was approved by the NHG DSRB (SDB-2024/0143) with a waiver of informed consent granted due to the retrospective nature of the study.

## RESULTS

Between 1 July 2022 and 30 September 2025, 39 patients were screened for inclusion. Six patients were excluded: two were reclassified as drug-susceptible following identification of silent rpoB mutation on whole-genome sequencing; one was found to have bedaquiline-resistance on drug-susceptibility testing (DST) after treatment initiation; one had partially completed a conventional long regimen prior to transition; and two had ongoing treatment at the time of analysis. The final cohort comprised 33 patients who received BPaLM/BPaL as part of routine care of RR/MDR-TB (Figure).

**Figure.**
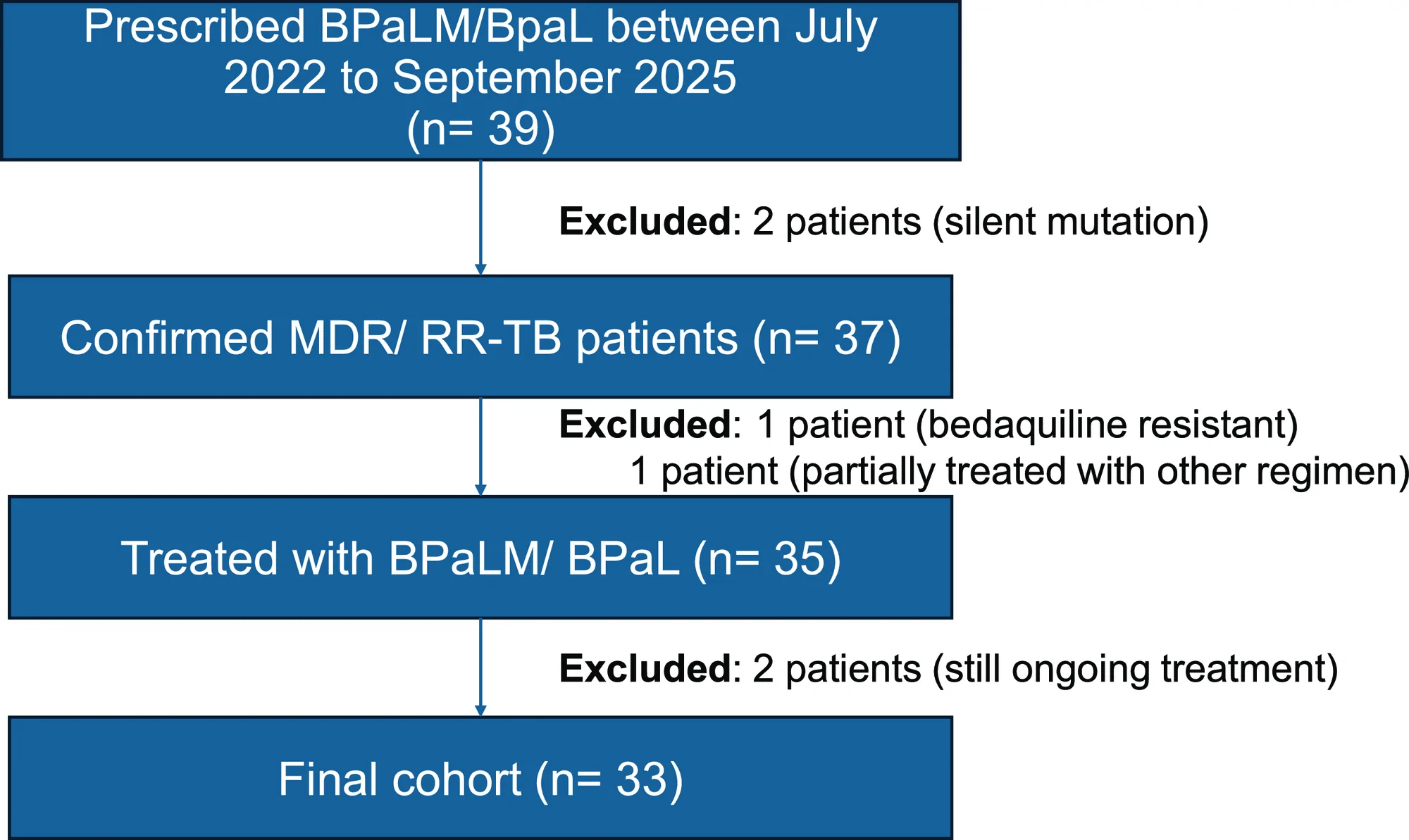
Flow diagram of patient screening and eligibility assessment. BPaLM/BPaL = bedaquiline, pretomanid, and linezolid with or without moxifloxacin; MDR/RR-TB = multidrug-resistant and rifampicin-resistant TB.

The median age was 39 years (IQR 28, 49). Fifteen patients (45.4%) were male, and 10 (30.3%) were Singapore-born. Five patients (15.2%) had a history of prior TB treatment – one patient was treated in our centre with first-line treatment, and the other four were treated by other centres.

### Disease characteristics and presentation

Among the cohort, 32 (97%) had pulmonary disease and were diagnosed from respiratory samples (31 from sputum, 1 from bronchoscopy). One patient had TB lymphadenitis with no pulmonary involvement. Fifteen patients (45.4%) were smear-negative, 8 (24.3%) had scanty or 1+ smear positivity, and 10 (30.3%) were highly smear-positive (smear 2+ to 4+). Twenty-nine (87.9%) were found to have RR on GeneXpert (Cepheid, Sunnyvale, CA) on initial diagnosis, while four were found to have RR only upon culture and DST confirmation. Thirty patients (90.9%) had positive mycobacterial cultures. DST identified rifampicin monoresistance in 6 patients (18.2%), MDR-TB in 22 patients (66.7%), and pre-XDR-TB (MDR-TB with additional resistance to any fluoroquinolone) in 2 patients (6.0%) (Table 1).

**Table 1.** Baseline characteristics and microbiology (n = 33).

Baseline characteristics	N (%)
Age (years)	39 (IQR 28, 49)
Male sex	15 (45.4)
Weight at diagnosis (kg)	54.5 (IQR 47, 61.2)
Singapore-resident	14 (42.4)
Singapore-born	10 (30.3)
Foreign-born	23 (69.7)
China	1 (3)
Indonesia	1 (3)
Malaysia	5 (15)
Myanmar	9 (27)
Philippines	5 (15)
Thailand	1 (3)
Vietnam	1 (3)
Comorbidities	
HIV	1 (3.0)
DM	3 (9.1)
Autoimmune condition	4 (12.1)
Cancer	2 (6.1)
CKD/ESRF	4 (12.1)
Liver cirrhosis	1 (3.0)
TB Microbiology	
Source	
Pulmonary	32 (97.0)
Extra-pulmonary	1 (3.0)
Smear	
0	15 (45.4)
Scanty	3 (9.1)
1+	5 (15.2)
2+	4 (12.1)
3+	4 (12.1)
4+	2 (6.0)
Xpert semi-quantitative load	
High	1 (3)
Medium	2 (6)
Low	7 (21.1)
Very low	8 (24.2)
Trace	1 (3)
Not available	14 (42.4)
Culture	
MTC positive	30 (90.9)
Drug susceptibility	
Unable to perform	3 (9.1)
Rifampicin monoresistant	6 (18.2)
Multidrug resistant	22 (66.7)
Pre-XDR	2 (6)

DM = diabetes mellitus; CKD/ESRF = chronic kidney disease/end-stage renal failure; MTC = *Mycobacterium tuberculosis* complex; pre-XDR = multidrug-resistant TB with additional resistance to any fluoroquinolone.

Patients presented either from screening programmes such as health screening or pre-employment screening (15 patients, 45.5%), incidental radiographic findings performed for reasons unrelated to TB (5 patients, 15.2%), or symptomatic presentation (13 patients, 39.4%). Among those detected through screening or incidental imaging, only 2 of 20 patients (10%) reported symptoms. Overall, asymptomatic disease was present in 18 patients (54.5%). Smear positivity was more common among symptomatic patients than asymptomatic patients (73.3% vs. 38.9%; odds ratio 0.23, 95% confidence interval: 0.05–1.02; *P* = 0.048).

### Treatment outcomes

The majority of patients (63.6%) were transitioned to BPaLM/BPaL after initial treatment with either first- or second-line regimens. This was because other centres had initiated alternative regimens before referring the patient to NTBCC, or programmatic factors such as the initial logistical constraints due to phased implementation of BPaLM/BPaL in the early study period, or delayed recognition of RR/MDR-TB. Thirteen patients (29.4%) received first-line treatment prior to BPaLM/BPaL initiation at a median duration of 4 days (IQR 2, 29) while eight patients (24.2%) received other second line regimens at a median duration of 25 days (IQR 8, 58) – see Table 2.

**Table 2.** Treatment characteristics and outcomes.

Treatment characteristics and outcome	N (%)
BPaLM/BPaL initiated at start	12 (36.4)
First-line regimen initiated before BPaLM/BPaL	13 (29.4)
Alternative second line regimen initiated before BPaLM/BPaL	8 (24.2)
Reasons for non-initiation of BPaLM/BPaL upfront (N = 21)	
Prior treatment at referring centre	3 (14.3)
Initial BPaLM/BPaL unavailability	5 (23.8)
Rifampicin resistance not known at baseline	13 (61.9)
Treatment completion status (N = 33)	
Completed treatment	16 (48.5)
Left country	17 (51.2)
Post-treatment follow-up (N = 16)	
Completed 2-year follow-up	5 (31.3)
Ongoing follow-up > 1 year	8 (50)
Ongoing follow-up < 1 year	3 (18.8)
Relapse	
Microbiologically confirmed relapse	0

BPaLM/BPaL = 6-month treatment of bedaquiline, pretomanid, and linezolid with or without moxifloxacin.

Sixteen patients (48.5%) completed BPaLM/BPaL treatment in Singapore, all of whom achieved treatment success. Out of the 16 patients who completed treatment in Singapore, 5 had rifampicin-monoresistant TB while 11 had MDR-TB. All patients who completed treatment were enrolled in routine post-treatment surveillance with 6-monthly chest radiography for 2 years; no relapses were observed during available follow-up. Seventeen patients (51.2%) transferred out of Singapore to continue treatment in their home countries; among these patients, the median duration of BPaLM/BPaL received locally was 96 days (IQR 69, 99).

Eleven patients (33.3%) had negative sputum cultures at the time of BPaLM/BPaL initiation. Among the remaining 22 patients, all achieved culture conversion with a median time to conversion of 49 days (IQR 28, 58.8).

### Adverse drug reactions

Adverse drug reactions (ADRs) requiring regimen modification occurred in 21 patients (63.6%), of which 13 patients (61.9%) had one ADR, 5 patients (23.8%) had two ADRs, and 3 patients (14.3%) had three ADRs (Table 3). The most frequently implicated drug was moxifloxacin which was discontinued in 14 patients (42.4%) for the following reasons: QTc prolongation (n = 6; median onset 41.5 days [IQR 17.3, 56]), gastrointestinal intolerance (n = 4; median onset 93.5 days [IQR 44.3, 196]), hepatotoxicity (n = 3; median onset 33 days [IQR 24.5, 34]), and rash (n = 1; onset at 28 days). Two patients had moxifloxacin discontinued upon confirmation of pre-XDR-TB on drug-susceptible testing.

**Table 3.** Adverse drug reactions and treatment modifications during BPaLM/BPaL therapy.

Adverse events and treatment modifications	N (%)
Total number of adverse drug reactions**^A^**	33
Patients with ≥1 adverse drug reaction	21 (63.6)
Patients with ≥2 adverse drug reactions	8 (24.2)
Patients with ≥3 adverse drug reactions	3 (9.0)
Drug implicated in adverse reactions**^A^**	
Bedaquiline	1 (3.0)
Linezolid	12 (36.4)
Moxifloxacin	14 (42.4)
Multiple drugs	6 (18.2)
Treatment modification due to toxicity	
Bedaquiline dose reduction	1 (3.0)
Linezolid dose reduction	11 (33.3)
Linezolid discontinuation	1 (3.0)
Moxifloxacin discontinuation	14 (42.4)
Temporary drug holiday	6 (18.2)
Most common adverse reaction categories	
Haematological abnormalities	9 (27.3)
Gastrointestinal intolerance	7 (21.2)
Hepatotoxicity	4 (12.1)
QTc prolongation	8 (24.2)
Neuropathy	4 (12.1)
Rash	1 (3.0)

BPaLM/BPaL = bedaquiline, pretomanid, and linezolid with or without moxifloxacin.

A More than one drug may be implicated per patient.

Linezolid dose reduction from 600 mg once daily to 600 mg thrice weekly or 300 mg once daily was required in 11 patients (33.3%) with one patient eventually requiring permanent cessation. Linezolid adverse reactions were predominantly due to haematological toxicity (n = 8; median onset 37.5 days [IQR 19, 73.5]), with neuropathy accounting for three cases (median onset 154 days [IQR 84, 154]).

Six patients required temporary cessation of all TB medications (drug holiday), with a median duration of cessation of 6 days (IQR 5, 7). This was in view of gastrointestinal intolerance (n = 3; median onset 79 days [IQR 49.5, 88.5]), QTc prolongation (n = 1; onset 61 days), neutropenia (n = 1; onset 47 days), and hepatotoxicity (n = 1; onset 112 days).

One patient required bedaquiline dose reduction to 100 mg OM at 62 days due to QTc prolongation.

In exploratory analyses, older age was more frequently observed among patients who required drug holiday. Patients who required a drug holiday were older than those who did not (median age 54.5 vs. 35 years, *P* = 0.028). No such association was found for patients who required moxifloxacin cessation (median age 44.5 vs. 34 years, *P* = 0.243) or linezolid dose reduction (median age 49 vs. 37.5 years, *P* = 0.504). It was also observed that linezolid dose reduction was significantly more frequent among patients with chronic kidney disease or end stage renal disease (CKD/ESRD) compared to those without renal impairment (4/4 [100%] vs. 6/29 [20.7%], *P* = 0.005). Temporary treatment cessation (drug holiday) was likewise more common in patients with CKD/ESRD than in those without renal impairment (3/4 [75%] vs. 3/29 [10.3%], *P* = 0.014). No statistically significant associations were observed between ADRs and people with diabetes mellitus (DM) or cirrhosis, although a non-significant trend towards increased treatment interruption and moxifloxacin cessation was seen among patients with DM. In contrast, no significant associations were identified between baseline comorbidities and moxifloxacin cessation overall.

## DISCUSSION

In this national cohort, BPaLM/BPaL demonstrated excellent early effectiveness in routine clinical practice. All patients who completed treatment locally achieved treatment success, despite frequent regimen modifications due to ADRs. These findings show that while BPaLM/BPaL regimens are highly effective, their successful implementation in routine care requires active monitoring for toxicity and flexible regimen management. ADRs requiring regimen modification occurred in 21 out of 33 patients (63.4%), reflecting cumulative toxicity of the drugs in the BPaLM/BPaL regimens. While clinical trials have established the efficacy of BPaLM/BPaL under controlled conditions, our findings highlight the realities of programmatic delivery, where gastrointestinal intolerance, linezolid-related toxicity, and cardiotoxic effects associated with moxifloxacin frequently necessitated treatment modification.

Moxifloxacin was the most frequently discontinued drug, with cessation occurring in 14 patients (42.4%), most commonly due to QTc prolongation (6/14) and gastrointestinal intolerance (6/14). This pattern likely reflects both the cumulative cardiotoxic risk of moxifloxacin when combined with bedaquiline and programmatic prioritisation within BPaLM/BPaL regimens, in which moxifloxacin is often considered the most readily modifiable component. This approach is supported by evidence from the Nix-TB trial,^4^ which demonstrated high efficacy with BPaL, supporting the feasibility of maintaining treatment effectiveness despite excluding fluoroquinolones.

Linezolid-related toxicity emerged as a major constraint in real-world implementation. In our cohort, 11 patients (33.3%) required linezolid dose reduction most commonly due to haematological toxicity and peripheral neuropathy. Of these, 10 were successfully managed with dose reduction alone, while one required permanent discontinuation of linezolid at 24 weeks due to anaemia, despite dose reduction to 300 mg daily after 12 weeks. The rate of linezolid-related toxicity in our cohort was higher than data from prior clinical trials such as ZeNIX,^5^ where linezolid dose modification was only required in 13% of the patients dosed using linezolid 600 mg daily for 26 weeks. While linezolid remains a cornerstone of contemporary RR/MDR-TB regimens, its dose-dependent haematological and neurological adverse effects frequently necessitate dose reduction or interruption.^15^ Current ATS/IDSA guidelines^16^ recommend therapeutic drug monitoring (TDM) to identify patients with elevated trough levels, which are associated with increased risk of toxicity, and to guide dose adjustments. However, in many programmatic settings, universal TDM is resource intensive and difficult to implement. Our experience suggests a pragmatic, risk-stratified approach to linezolid toxicity monitoring, prioritising older patients and those with CKD/ESRD, rather than routine universal TDM, particularly in resource-constrained settings.

Another important consideration, given the high rates of adverse reactions to linezolid, is empirical dose reduction of linezolid. In the TB-PRACTECAL^6^ trial, linezolid dosed at 600 mg for 16 weeks followed by 300 mg for 8 weeks showed 88% treatment success. A recent individual patient data meta-analysis^17^ of 945 patients further supported this approach, demonstrating that the TB-PRACTECAL linezolid dosing was associated with high treatment success (90.4%) and fewer adverse reactions compared to continuous 600 mg dosing. In addition, a randomised control trial^18^ performed on patients with pre-XDR-TB and non-tolerant or non-responsive MDR-TB treated with BPaL showed that reducing the dose of linezolid from 600 mg daily to 300 mg daily at 9–13 weeks was non-inferior to the standard 600 mg daily dose for 26 weeks but with significantly less neuropathy. In our cohort, 11 patients required linezolid dose reduction at a median time of 6.7 weeks, substantially earlier than the scheduled dose reduction in TB-PRACTECAL. Of these 11 patients, 9 successfully completed treatment in Singapore while 2 left the country. These findings suggest that linezolid toxicity occurs early in the treatment course and may necessitate earlier, individualised dose reduction. Close monitoring is therefore essential, with a low threshold for dose adjustment to balance tolerability and treatment efficacy.

The findings of our study add to emerging evidence on shortened 6-month regimens for MDR-TB. In 2024, the WHO endorsed alternative 6-month regimen based on bedaquiline, delamanid, and linezolid in combination with either levofloxacin or clofazimine or both (BDLLfxC)^19^ based on the results of the BEAT-TB trial.^20^ The subsequent endTB-Q trial^21^ also showed favourable pre-XDR-TB outcomes with bedaquiline, delamanid, linezolid, and clofazimine, although efficacy was lower in patients with extensive disease, highlighting that shorter regimens may not be suitable for all patients and that treatment should be individualised according to disease extent. A consistent feature across these shortened regimens is the central role of linezolid, which remains one of the most effective but also most toxicity-prone drugs. As demonstrated by our findings, there remains a need to remain vigilant in the real-world setting to monitor for linezolid toxicity, as the need for dose adjustment can occur earlier than the trial-specified dose adjustments. There is also a need to evaluate the efficacy of these regimens should early linezolid dose adjustments be required.

Although our study demonstrated excellent effectiveness for the treatment of RR/MDR-TB, our cohort excluded patients with disseminated TB in accordance with the WHO recommendations.^13^ As highlighted in a recent review article^22^ on a 6-month regimens for drug-resistant TB, there are limited real-world data for difficult-to-treat TB such as central nervous system TB, musculoskeletal TB, and disseminated TB, where drug penetration may be suboptimal and longer durations of BPaLM/BPaL and BDLLfxC may be required. In such cases, early linezolid toxicity may limit the effectiveness of these regimens and individualisation is required to balance the duration of treatment needed to achieve cure while minimising drug toxicity.

Finally, from our experience, shorter regimens do not eliminate the need for robust treatment support. At NTBCC, treatment is at no cost to the patient and there are established directly observed therapy (DOT) programmes. Early access to specialist clinical review and social support measures likely facilitated both adherence and timely management of adverse events. All patients receive inpatient DOT during the initiation of BPaLM/BPaL, and upon discharge continue outpatient DOT at their nearest primary health care clinics. These systems likely mitigate the clinical impact of ADRs and frequent regimen modifications and contribute to favourable early treatment outcomes.

## CONCLUSION

In a real-world TB centre, BPaLM/BPaL achieved excellent early effectiveness for RR/MDR-TB treatment but requires active and flexible regimen management due to frequent ADRs. Successful implementation depends not only on regimen potency but also on anticipatory toxicity monitoring and strong treatment, financial, and psychosocial support systems. These findings provide practical insights for TB programmes adopting short-course all-oral regimens under routine care conditions.

## References

[1] World Health Organization. Global tuberculosis report, 2025. Geneva: World Health Organization, 2025.

[2] Lan Z, Drug-associated adverse events in the treatment of multidrug-resistant tuberculosis: an individual patient data meta-analysis. Lancet Respir Med. 2020;8(4):383-394. 32192585 10.1016/S2213-2600(20)30047-3 PMC7384398

[3] Van Deun A, World Health Organization 2018 treatment guidelines for rifampicin-resistant tuberculosis: uncertainty, potential risks and the way forward. Int J Antimicrob Agents. 2020;55(1):105822. 31626907 10.1016/j.ijantimicag.2019.10.003

[4] Conradie F, Treatment of highly drug-resistant pulmonary tuberculosis. New Engl J Med. 2020;382(10):893-902. 32130813 10.1056/NEJMoa1901814 PMC6955640

[5] Conradie F, Bedaquiline–pretomanid–linezolid regimens for drug-resistant tuberculosis. New Engl J Med. 2022;387(9):810-823. 36053506 10.1056/NEJMoa2119430 PMC9490302

[6] Nyang’wa B-T, Short oral regimens for pulmonary rifampicin-resistant tuberculosis (TB-PRACTECAL): an open-label, randomised, controlled, phase 2B-3, multi-arm, multicentre, non-inferiority trial. Lancet Respir Med. 2024;12(2):117-128. 37980911 10.1016/S2213-2600(23)00389-2

[7] Goodall RL, Evaluation of two short standardised regimens for the treatment of rifampicin-resistant tuberculosis (STREAM stage 2): an open-label, multicentre, randomised, non-inferiority trial. Lancet. 2022;400(10366):1858-1868. 36368336 10.1016/S0140-6736(22)02078-5 PMC7614824

[8] Sangsayunh P, The use of BPaL containing regimen in the MDR/PreXDR TB treatments in Thailand. J Clin Tuberc Other Mycobact Dis. 2024;34:100408. 38225943 10.1016/j.jctube.2023.100408 PMC10788258

[9] Myrzaliev B, Experiences in the introduction of bedaquiline pretomanid linezolid for drug-resistant tuberculosis in Kyrgyzstan. J Clin Tuberc Other Mycobact Dis. 2024;36:100472. 39175915 10.1016/j.jctube.2024.100472 PMC11338960

[10] Wares DF, Introducing BPaL: experiences from countries supported under the LIFT-TB project. PLoS One. 2024;19(11):e0310773. 39561206 10.1371/journal.pone.0310773 PMC11575791

[11] Khan MA, Experience of piloting BPaLM/BPaL for DR-TB care at selected sites in Pakistan. IJTLD Open 2024;1(11):508-515. 39544887 10.5588/ijtldopen.24.0369 PMC11558782

[12] Dahl VN, Effectiveness of the bedaquiline, pretomanid, and linezolid regimen, with or without moxifloxacin, for drug-resistant tuberculosis in Ukraine under programmatic conditions. Clin Infect Dis. 2025;81(4):853-856. 40280886 10.1093/cid/ciaf214 PMC12596402

[13] World Health Organization. WHO consolidated guidelines on tuberculosis. Module 4: treatment - drug-resistant tuberculosis treatment, 2022 update. 32603040

[14] Nguyen NL, New treatment outcome definitions for drug-susceptible and drug-resistant tuberculosis: update from World Health Organization. Eur. Respir. J. 2021;58(2):2011804. 10.1183/13993003.00804-2021 34413124

[15] Hasan T, The safety and tolerability of linezolid in novel short-course regimens containing bedaquiline, pretomanid, and linezolid to treat rifampicin-resistant tuberculosis: an individual patient data meta-analysis. Clin Infect Dis. 2024;78(3):730-741. 37874021 10.1093/cid/ciad653 PMC10954324

[16] Saukkonen JJ, Updates on the treatment of drug-susceptible and drug-resistant tuberculosis: an official ATS/CDC/ERS/IDSA clinical practice guideline. Am J Respir Crit Care Med. 2025;211(1):15-33. 40693952 10.1164/rccm.202410-2096ST PMC11755361

[17] Kwak N, Optimal dosing and duration of linezolid for the treatment of multidrug-resistant and rifampicin-resistant tuberculosis: an individual patient data meta-analysis. Eur Respir J. 2025;66(2):2500315. 40675768 10.1183/13993003.00315-2025 PMC12371315

[18] Padmapriyadarsini C, Effectiveness and safety of varying doses of linezolid with bedaquiline and pretomanid in treatment of drug-resistant pulmonary tuberculosis: open-label, randomized clinical trial. Clin Infect Dis. 2024;79(6):1375-1385. 39194339 10.1093/cid/ciae388

[19] Key updates to the treatment of drug-resistant tuberculosis. 2024. 10.2471/B09123.

[20] Conradie F, A pragmatic trial of a 6-month strategy for rifampicin-resistant tuberculosis. N Engl J Med. 2026;394(24):2429-2439. 42341301 10.1056/NEJMoa2503687

[21] Guglielmetti L, Bedaquiline, delamanid, linezolid, and clofazimine for rifampicin-resistant and fluoroquinolone-resistant tuberculosis (endTB-Q): an open-label, multicentre, stratified, non-inferiority, randomised, controlled, phase 3 trial. Lancet Respir Med. 2025;13(9):809-820. 40683298 10.1016/S2213-2600(25)00194-8 PMC13508822

[22] Davoli C, Can 6-month long regimens become the standardized treatment for MDR-TB globally?. Int J Infect Dis. 2025;160:108065. 40953688 10.1016/j.ijid.2025.108065

